# Traumatic Brain Injury and All-Cause and Dementia-Related Mortality in the Framingham Heart Study

**DOI:** 10.1001/jamanetworkopen.2025.55138

**Published:** 2026-01-30

**Authors:** Rebecca Burton, Shruti Durape, Eden Price, Kurtis Chien-Young, Prajakta Joshi, Eukyung Yhang, Yulin Liu, Sherral Devine, Ashita S. Gurnani, Ting Fang Alvin Ang, Douglas I. Katz, Michael L. Alosco, Yorghos Tripodis, Rhoda Au, Kristen Dams-O’Connor, Jesse Mez

**Affiliations:** 1 Framingham Heart Study, Boston University Chobanian and Avedisian School of Medicine, Boston, Massachusetts; 2 Department of Psychology, The City College of New York, New York, New York; 3 Department of Neurology, Boston University Chobanian and Avedisian School of Medicine, Boston, Massachusetts; 4 Boston University Alzheimer’s Disease Research Center, Boston University Chobanian and Avedisian School of Medicine, Boston, Massachusetts; 5 Boston University Chronic Traumatic Encephalopathy Center, Boston University Chobanian and Avedisian School of Medicine, Boston, Massachusetts; 6 Department of Neurology, Yale School of Medicine, New Haven, Connecticut; 7 Department of Biostatistics, Boston University School of Public Health, Boston, Massachusetts; 8 Department of Anatomy and Neurobiology, Boston University Chobanian and Avedisian School of Medicine, Boston, Massachusetts; 9 Braintree Rehabilitation Hospital, Braintree, Massachusetts; 10 Department of Epidemiology, Boston University School of Public Health, Boston, Massachusetts; 11 Department of Rehabilitation and Human Performance, Icahn School of Medicine at Mount Sinai, New York, New York; 12 Department of Neurology, Icahn School of Medicine at Mount Sinai, New York, New York

## Abstract

**Importance:**

Traumatic brain injury (TBI) has been associated with mortality and chronic conditions, including dementia. However, research is limited regarding the disease process by which TBI leads to long-term mortality.

**Objective:**

To assess TBI incidence over decades and its association with long-term all-cause and dementia-related mortality among participants in the Framingham Heart Study (FHS), with the hypothesis that the association would be largely attributable to dementia-related mortality.

**Design, Setting, and Participants:**

This cohort study used data from the original and offspring cohorts of the FHS, a multigenerational, community-based cohort study conducted from 1948 to 2022. Participants with TBI were matched 1:3 to unexposed participants based on birth year, sex, and generational cohort. TBI data were collected from comprehensive medical record review and study examination visit records from the time of enrollment until death or conclusion of data collection. Data analysis was performed from February 1, 2023, to November 1, 2024.

**Exposure:**

Traumatic brain injury.

**Main Outcomes and Measures:**

The primary outcomes assessed were all-cause and dementia-related mortality. Time-to-event analyses were conducted using data from the FHS original and offspring cohorts.

**Results:**

This study included 10 333 FHS participants: 5209 from the original cohort (mean [SD] age at enrollment, 44 [9] years; 55.2% female; mean follow-up, 35 [15] years) and 5124 from the offspring cohort (mean [SD] age at enrollment, 36 [11] years; 51.5% female; mean follow-up, 39 [11] years). A total of 886 participants (17.0%) in the original cohort and 1243 (24.3%) in the offspring cohort experienced at least 1 TBI, with incidence rates of 7.02 (95% CI, 6.63-7.40) and 9.11 (95% CI, 8.70-9.51) TBI events per 1000 person-years and a mean (SD) age at TBI of 74 (16) and 71 (15) years, respectively. Falls were the most common mechanism of TBI in both the original (65.4%) and offspring (82.8%) cohorts. TBI was associated with all-cause mortality (hazard ratio [HR], 1.15 [95% CI, 1.06-1.26]) in a dose-dependent fashion with TBI severity (mild: HR, 1.06 [95% CI, 0.96-1.16]; moderate to severe: HR, 1.82 [95% CI, 1.48-2.25]). TBI was also associated with dementia-related mortality in a dose-dependent fashion (mild TBI: HR, 1.60 [95% CI, 1.31-1.97]; moderate to severe TBI: HR, 3.67 [95% CI, 2.31-5.80]) but not with non–dementia-related mortality.

**Conclusions and Relevance:**

In this study of FHS community-based original and offspring cohorts followed up over 7 decades, most TBIs occurred late in life and were related to falls. TBI was associated with an increased risk of long-term, all-cause mortality in a dose-dependent fashion, with dementia-related mortality largely contributing to this association. Preventing falls to reduce TBI could have important implications for dementia and mortality.

## Key Points

**Question:**

What is the incidence of traumatic brain injury (TBI), and is TBI associated with long-term all-cause and dementia-related mortality?

**Findings:**

In this cohort study of 10 333 participants in the Framingham Heart Study followed from 1948 to 2022, TBI most often occurred in late life due to falls, with an incidence of 7.02 (original cohort) and 9.11 (offspring cohort) TBI events per 1000 person-years. TBI was associated with increased all-cause and dementia-related mortality but not with non–dementia-related mortality.

**Meaning:**

The findings of this study suggest that preventing falls to reduce TBI could have important implications for dementia and mortality.

## Teaser

This cohort study uses data from the Framingham Heart Study original and offspring cohorts followed from 1948 to 2022 to assess whether traumatic brain injury (TBI) incidence is associated with long-term all-cause and dementia-related mortality.

## Introduction

Traumatic brain injury (TBI), defined as an alteration in brain functioning or brain pathology due to an external force, is a leading cause of disability and death in the US.^1,2^ An estimated 2.8 to 3 million US residents are diagnosed with a TBI annually, and approximately 3.2 to 5.3 million US residents are currently living with a TBI-related disability.^2,3,4,5^ In 2017, 25% of all injury-related deaths in the US involved TBIs, and in 2021, 190 Americans died from TBI-related injuries each day.^6,7^ Previous studies investigating TBI and neurodegenerative diseases have found a history of TBI to be associated with the development of dementia, although research is inconsistent regarding underlying etiology.^8,9,10,11,12,13^ Additionally, compared with individuals without TBI, those hospitalized with mild TBI had a greater risk of death within 15 years after hospitalization, and those with moderate or severe TBI had an increased risk of death with a decrease in life expectancy by 7 years.^14,15,16^ With these findings, researchers have argued for TBI to be considered a chronic condition or a silent epidemic.^17,18,19^

Although research has begun to characterize TBI as a chronic condition associated with long-term consequences such as dementia and mortality, traditional TBI treatments and care models prioritize acute care.^17,20^ Research examining TBI as a risk factor for initiating or accelerating neurodegenerative disease is mixed, and studies investigating the connection between history of TBI and mortality are limited.^14,21,22,23,24^ Previous research frequently relied on death certificates (on which dementia as a contributory cause is often omitted^25,26,27^), eliminating the possibility that dementia may lead to premature mortality following TBI.^28,29,30,31^ Additionally, population-based studies have relied on data collected from either hospital admissions or self-report alone, thereby limiting their characterization of TBI. Further systematic, scientifically rigorous research examining TBI in relation to neurodegenerative disease and mortality is limited.^17,21,22,23,24^

This study used data collected between 1948 and 2022 from medical record review, self-report, and study examination visit records of the original and offspring cohorts of the Framingham Heart Study (FHS) to examine the incidence of TBI over time and across time- and age-decades, including reporting setting and mechanism of injury. Additionally, among those who survived at least 1 year post injury, we investigated the risk of long-term mortality associated with TBI, and we hypothesized that dementia-related mortality would largely contribute to the association. Finally, we examined the association between severity and number of TBIs and long-term mortality.

## Methods

### Study Design and Participants

The FHS is a longitudinal, community-based cohort study.^21^ Analyses for this cohort study included participants from the first 2 FHS generation cohorts, including the original cohort with enrollment beginning in 1948 and the offspring cohort with enrollment beginning in 1971.^31,32^ Participants were characterized longitudinally until death, with comprehensive examinations at study visits and surveillance questionnaires between examinations. At the conclusion of this study’s data collection, the original cohort had undergone 32 examinations that were conducted every 2 years and were concluded in 2014, and the offspring cohort had undergone 10 examinations that were conducted every 4 years.^31^ Participants also released medical records to the FHS from hospital visits, nursing home facilities and hospice care, urgent care centers, and outpatient and primary care visits from the time of recruitment until death. Additional information, can be found in the studies by Dawber et al^32^ and Feinleib et al.^33^ The Boston University Medical Center institutional review board approved the study protocols and consent forms. All participants provided written informed consent. The study followed the Strengthening the Reporting of Observational Studies in Epidemiology (STROBE) reporting guideline.

### TBI Data Collection and Adjudication

TBI data came from both comprehensive medical record review and self-report TBI questionnaires. Medical records were requested on an ongoing basis from the time of enrollment, during each core visit, and during annual health status updates. Study personnel reviewed every FHS and medical record across all participants from both cohorts to find instances of head impact, TBI symptomatology, confounding factors, neuroimaging reports, and postinjury sequelae. Study personnel conducting medical record review were extensively trained, and strict quality control procedures were implemented. When questions or discrepancies arose, cases were elevated to senior personnel, the principal investigator (J.M.), or both for resolution. Self-report TBI questions were administered at a subset of core examinations (original cohort: examinations 19, 22, and 23; and offspring cohort: examinations 7, 8, and 9). For a full description of this process, see the eMethods and eFigure 1 in Supplement 1.

Injuries were categorized as either (1) head injury but not meeting TBI criteria or (2) TBI, which was further categorized based on severity as mild, moderate, or severe. TBI severity was classified using modified American Congress of Rehabilitation Medicine and US Veterans Affairs/Department of Defense criteria (eTable 1 in Supplement 1).^34^

### Mortality

We monitored participants until death or conclusion of the follow-up period (August 15, 2022). Death reviews were conducted using data from hospital and emergency department records, imaging and laboratory reports, physicians’ notes, death certificates, and autopsy or medical examiners’ reports.^35^ Diagnoses of dementia were assigned by a dementia review panel, including neurologists and neuropsychologists, using *Diagnostic and Statistical Manual of Mental Disorders* (Fourth Edition) (*DSM-IV*) criteria.^31,36,37^ We categorized causes of death as dementia related and nondementia related. Dementia-related mortality included participants who were determined at death review to have died from other causes and were adjudicated as having dementia based on neuropsychological testing and gold-standard consensus adjudication using *DSM-IV* criteria.^31,37^ Dementia-related mortality was further subdivided into pure Alzheimer disease (AD)–related mortality (based on an adjudicated diagnosis of National Institute of Neurological and Communicative Disorders and Stroke [NINCDS] probable AD) and other dementia-related mortality (those with dementia-related mortality but not meeting NINCDS probable AD criteria).^38^ Nondementia-related mortality included participants who were determined to have died from cardiovascular disease (CVD), cerebrovascular accident (CVA), cancer, other causes (without having been adjudicated to have dementia), or unknown causes.

### Covariates

Additional factors (CVD, current smoking, hypertension, type 2 diabetes, and education level) were extracted from the examination closest to the TBI event and at the same time for the matched unexposed group. CVD was defined as the presence of any of the following: atrial fibrillation, stroke, or coronary heart disease, which were assessed by physicians who reviewed each participant’s study data and medical records systematically. Diagnoses of hypertension and type 2 diabetes were similarly assessed by physicians who systematically reviewed each participant’s study data and medical records. Detailed methods of the surveillance, evaluation, and diagnoses have been described elsewhere.^36^ Education was classified into 4 ordered levels: no high school, high school diploma, some college, and college degree. Smoking status was defined as having ever smoked.

### Statistical Analysis

TBI incidence was calculated in number of TBI events per 1000 person-years throughout the participant lifespan as well as across age- and time-decades and by mechanism of injury. Only TBI events and person-years occurring after participant enrollment were considered. A Poisson distribution was assumed to calculate 95% CIs for the TBI incidence rates.

To investigate associations between TBI and long-term mortality, participants with and without incident TBI were randomly matched on study cohort, birth year, and sex in a 1:3 ratio. Participants with TBI reported in the medical record as occurring prior to study enrollment (ie, prevalent TBI) were excluded. Because we were interested in long-term mortality, participants who died within a year of the TBI event were excluded. We used the same constraint for matches without TBI, who could not die within 1 year of the corresponding TBI. Missing values for covariates were imputed using multiple imputation by chained equations, creating 10 imputed datasets. All covariates informed imputation. Individual imputation models used logistic regression to impute ordered categorical variables. eTable 2 in Supplement 1 provides missingness rates.

For primary analyses, we estimated the association between TBI and all-cause mortality by occurrence and severity using Cox proportional hazards regression. The clustering induced by matching was handled in the Cox regression using a robust (sandwich) variance estimator clustered on the matched set that accounts for the within-set dependence without changing the point estimates. Due to the relatively small number of participants with moderate or severe TBI, we grouped them together. For the TBI group, person-time was considered to begin at the time of first TBI and at the equivalent time for the matched group. Analysis using the matched cohort and anchored follow-up at the time of TBI was conducted to maintain comparability between participants with and without TBI and to avoid including person-time before TBI in the unexposed group, potentially introducing a mixture of 2 distinct groups. Subsequently, we explored the association between TBI and dementia-related and non–dementia-related mortality using a cause-specific competing risk model. All models were adjusted for covariates, including education status, cigarette smoking, diabetes, hypertension, CVD, and stroke history. We tested the proportional hazards assumption using Schoenfeld residuals. The significance threshold for all models was established at 2-sided *P* < .05.

In sensitivity analyses, we repeated analyses only among participants with TBI prior to cognitive impairment onset and matched control participants (without cognitive impairment or TBI). We also repeated analyses only among participants with TBI after cognitive impairment onset and matched control participants (without TBI and with cognitive impairment). In addition, we repeated analyses excluding participants and their matches who died within 2 years of TBI. In secondary analyses, we evaluated the association between TBI and mortality stratified by sex and age at TBI (<60 and ≥60 years). For each subgroup analysis, we conducted formal tests of statistical interaction. To investigate the role of multiple TBIs among participants with TBI, we matched participants with a single TBI in a 2:1 ratio to those with multiple TBIs by study cohort, age at first TBI, and sex and examined the risk of all-cause and dementia-related mortality by number of TBIs (single vs multiple). This matching approach was chosen rather than comparing each group to those without TBI, because of marked age differences between those with single and multiple TBIs that would have otherwise been unaddressed.

We reran the cause-specific competing risk models for each cause of death rather than grouping the non–dementia-related causes together. Additionally, we reran the cause-specific competing risk models to further stratify dementia-related mortality into pure AD dementia-related mortality and other dementia-related mortality.

Data collection was performed using REDCap.^39^ Statistical analyses were performed using SAS, version 9.4 (SAS Institute Inc); R, version 3.3.1 (R Project for Statistical Computing); and Python, version 3.12.4 (Python Software Foundation). Data analysis was performed from February 1, 2023, to November 1, 2024.

## Results

Participant-level demographic data for the FHS original and offspring cohorts (N = 10 333) stratified by participants’ most severe TBI are summarized in Table 1. A total of 5209 participants (55.2% female and 44.8% male) from the original cohort and 5124 (51.5% female and 48.5% male) from the offspring cohort were followed up for a mean (SD) of 35 (15) and 39 (11) years, respectively. The mean (SD) age at enrollment was 44 (9) years for the original cohort and 36 (11) years for the offspring cohort. All 5209 participants (100%) from the original cohort and 2330 (45.5%) from the offspring cohort were deceased at the end of monitoring. A total of 886 participants (17.0%) in the original cohort and 1243 (24.3%) in the offspring cohort experienced at least 1 TBI within the study period. There were 3166 total TBIs, with 1264 TBIs in the original cohort and 1902 TBIs in the offspring cohort. Few TBIs were ascertained from self-report alone (32 of 3166 [1.0%]), with most ascertained from medical records alone (2660 of 3166 [84.0%]) or both medical records and self-report (474 of 3166 [15.0%]).

**Table 1.** Demographic Data for the Framingham Heart Study Original and Offspring Cohorts Stratified by Most Severe TBI Experienced^a^

Characteristic	Participants without TBI	Participants with TBI				All participants
		Worst TBI during study			Any TBI	
		Mild	Moderate	Severe		
**Original cohort**						
Participants	4323/5209 (83.0)	666/886 (75.2)	128/886 (14.4)	92/886 (10.4)	886/5209 (17.0)	5209/5209 (100)
Sex						
Female	2318/4323 (53.6)	429/666 (64.4)	84/128 (65.6)	42/92 (45.7)	555/886 (62.6)	2873/5209 (55.2)
Male	2005/4323 (46.4)	237/666 (35.6)	44/128 (34.4)	50/92 (54.3)	331/886 (37.4)	2336/5209 (44.8)
Age at enrollment, mean (SD) [range], y	45 (9) [28-74]	41 (8) [29-62]	40 (8) [30-59]	44 (10) [30-61]	41 (8) [29-62]	44 (9) [28-74]
Education						
No high school diploma	1804/4159 (43.4)	257/648 (39.7)	45/124 (36.3)	46/88 (52.3)	348/860 (40.5)	2152/5019 (42.9)
High school diploma	1214/4159 (29.2)	199/648 (30.7)	37/124 (29.8)	24/88 (27.3)	260/860 (30.2)	1474/5019 (29.4)
Some college	666/4159 (16.0)	125/648 (19.3)	20/124 (16.1)	8/88 (9.1)	153/860 (17.8)	819/5019 (16.3)
College degree	475/4159 (11.4)	67/648 (10.3)	22/124 (17.7)	10/88 (11.4)	99/860 (11.5)	574/5019 (11.4)
Smoking history	2511/4171 (60.2)	352/652 (54.0)	75/126 (59.5)	49/89 (55.1)	476/867 (54.9)	2987/5038 (59.3)
Diabetes	595/4323 (13.8)	107/666 (16.1)	17/128 (13.3)	12/92 (13.0)	136/886 (15.3)	731/5209 (14.0)
Hypertension	4081/4323 (94.4)	638/666 (95.8)	124/128 (96.9)	88/92 (95.7)	850/886 (95.9)	4931/5209 (94.7)
Coronary heart disease	1812/4323 (41.9)	253/666 (38.0)	41/128 (32.0)	26/92 (28.3)	320/886 (36.1)	2132/5209 (40.9)
Stroke	811/4323 (18.8)	125/666 (18.8)	41/128 (32.0)	17/92 (18.5)	183/886 (20.7)	994/5209 (19.1)
Age at death, mean (SD) [range], y	77 (13) [28-74]	85 (10) [49-110]	87 (13) [52-101]	81 (12) [29-62]	85 (11) [37-110]	78 (13) [32-110]
No. of total deaths	4270	659	128	92	879	5149
Cause of death						
Heart disease (coronary or other)	1318/4270 (30.9)	137/659 (20.8)	15/128 (11.7)	7/65 (10.8)	159/879 (18.1)	1477/5149 (28.7)
Cerebrovascular accident	300/4270 (7.0)	38/659 (5.8)	10/128 (7.8)	12/92 (13.0)	60/879 (6.8)	360/5149 (7.0)
Cancer	986/4270 (23.1)	102/659 (15.5)	18/128 (14.1)	4/92 (4.3)	124/879 (14.1)	1110/5149 (21.6)
Dementia related	445/4270 (10.4)	181/659 (27.5)	45/128 (35.2)	16/92 (17.4)	242/879 (27.5)	687/5149 (13.3)
Other	678/4270 (15.9)	108/659 (16.4)	27/128 (21.1)	48/92 (52.2)	183/879 (20.8)	861/5149 (16.7)
Unknown	543/4270 (12.7)	93/659 (14.1)	13/128 (10.2)	5/92 (5.4)	111/879 (12.6)	654/5149 (12.7)
**Offspring cohort**						
Participants	3881/5124 (75.7)	870/1243 (70.0)	221/1243 (17.8)	152/1243 (12.2)	1243/5124 (24.3)	5124/5124 (100)
Sex						
Female	1983/3881 (51.1)	463/870 (53.2)	122/221 (55.2)	73/152 (48.0)	658/1243 (52.9)	2641/5124 (51.5)
Male	1898/3881 (48.9)	407/870 (46.8)	99/221 (44.8)	79/152 (52.0)	585/1243 (47.1)	2483/5124 (48.5)
Age at enrollment, mean (SD) [range], y	36 (11) [5-70]	38 (10) [10-63]	39 (10) [15-64]	39 (10) [13-67]	38 (10) [10-67]	36 (11) [5-70]
Education						
No high school diploma	215/3215 (6.7)	45/790 (5.7)	17/201 (8.5)	15/125 (12.0)	77/1116 (6.9)	292/4331 (6.7)
High school diploma	906/3215 (28.2)	248/790 (31.4)	71/201 (35.3)	32/125 (25.6)	351/1116 (31.5)	1257/4331 (29.0)
Some college	825/3215 (25.7)	190/790 (24.1)	57/201 (28.4)	32/125 (25.6)	279/1116 (25.0)	1104/4331 (25.5)
College degree	1269/3215 (39.5)	307/790 (38.9)	56/201 (27.9)	46/125 (36.8)	409/1116 (36.6)	1678/4331 (38.7)
Smoking history	1992/3878 (51.4)	381/870 (51.4)	95/221 (43.0)	62/152 (40.8)	538/1243 (43.3)	2530/5121 (49.4)
Diabetes	624/3881 (16.1)	174/870 (20.0)	47/221 (21.3)	31/152 (20.4)	252/1243 (20.3)	876/5124 (17.1)
Hypertension	2945/3881 (75.9)	718/870 (82.5)	188/221 (85.1)	126/152 (82.9)	1032/1243 (83.0)	3977/5124 (77.6)
Coronary heart disease	762/3881 (19.6)	197/870 (22.6)	57/221 (25.8)	44/152 (28.9)	298/1243 (24.0)	1060/5124 (20.7)
Stroke	297/3881 (7.6)	94/870 (10.8)	34/221 (15.4)	23/152 (15.1)	151/1243 (12.1)	448/5124 (8.7)
*APOE* ε4 allele (either 1 or 2 copies)	650/3006 (21.6)	150/756 (19.8)	42/196 (21.4)	22/120 (18.3)	214/1072 (20.0)	864/4078 (21.2)
Age at death, mean (SD) [range], y	71 (13) [20-100]	79 (10) [29-100]	81 (10) [49-94]	74 (15) [16-95]	79 (11) [16-100]	73 (13) [28-74]
No. of total deaths	1729	382	122	104	608	2337
Cause of death						
Heart disease (coronary or other)	336/1725 (19.5)	69/378 (18.3)	28/121 (23.1)	15/104 (14.4)	112/603 (18.6)	448/2328 (19.2)
Cerebrovascular accident	62/1725 (3.6)	15/378 (4.0)	10/121 (8.3)	8/104 (7.7)	33/603 (5.5)	95/2328 (4.1)
Cancer	641/1725 (37.2)	93/378 (24.6)	16/121 (13.2)	17/104 (16.4)	125/603 (20.7)	766/2328 (32.9)
Dementia related	150/1725 (8.7)	91/378 (24.1)	34/121 (28.1)	23/104 (22.1)	148/603 (24.5)	298/2328 (12.8)
Other	383/1725 (22.2)	73/378 (19.3)	22/121 (18.2)	36/104 (34.6)	131/603 (21.7)	514/2328 (22.1)
Unknown	153/1725 (8.9)	37/378 (9.8)	11/121 (9.1)	5/104 (4.8)	53/603 (8.8)	206/2328 (8.8)

Abbreviations: *APOE*, apolipoprotein E; TBI, traumatic brain injury.

^a^ Unless indicated otherwise, values are presented as No./total No. (%) of participants. For cause of death, percentages are based on total deaths.

Characteristics of TBIs stratified by severity experienced by the original and offspring cohorts are summarized in Table 2. The mean (SD) age at TBI was 74 (16) years and 71 (15) years for the original and offspring cohorts, respectively. For the original and offspring cohorts, the most common TBI setting was residential (56.6% and 60.6%), and falls were the most common mechanism of TBI (65.4% and 82.8%).

**Table 2.** Prevalence and Characteristics of TBIs Experienced by the Framingham Heart Study Original and Offspring Cohorts^a^

Characteristic	TBI severity			All TBIs
	Mild TBI	Moderate TBI	Severe TBI	
**Original cohort**				
TBIs	993/1264 (78.6)	173/1264 (13.7)	98/1264 (7.8)	1264/1264 (100)
Sex				
Female	665/993 (67.0)	109/173 (63.0)	46/98 (46.9)	820/1264 (64.9)
Male	328/993 (33.0)	64/173 (37.0)	52/98 (53.1)	444/1264 (35.1)
Age at injury, mean (SD) [range], y	73 (17) [31-103]	81 (14) [41-102]	79 (12) [38-97]	74 (16) [31-103]
Setting of injury				
Residential	397/765 (51.9)	113/151 (74.8)	60/92 (65.2)	570/1008 (56.6)
Recreational	27/765 (3.5)	4/151 (2.6)	4/92 (4.3)	35/1023 (3.4)
Transportation	301/765 (39.4)	19/151 (12.6)	24/92 (26.1)	344/1008 (34.1)
Occupational	14/765 (1.8)	2/151 (1.3)	2/92 (2.2)	18/1008 (1.8)
Organized sports	0	1/151 (0.7)	0	1/1008 (0.1)
Military	4/765 (0.5)	2/151 (1.3)	1/92 (1.1)	7/1008 (0.7)
Other	22/765 (2.9)	10/151 (6.6)	1/92 (1.1)	33/1008 (3.3)
Mechanism of injury				
Acceleration or deceleration	46/939 (4.9)	2/163 (1.2)	1/90 (1.1)	49/1192 (4.1)
Head struck by or against an object	68/939 (7.2)	10/163 (6.1)	3/90 (3.3)	81/1192 (6.8)
Fall with trauma to the head	574/939 (61.1)	139/163 (85.3)	66/90 (73.3)	779/1192 (65.4)
Force generated by a blast or explosion	9/939 (1.0)	1/163 (0.6)	0	10/1192 (0.8)
Foreign body penetrating the brain	1/939 (0.1)	1/163 (0.6)	3/90 (3.3)	5/1192 (0.4)
Motor vehicle crash, mechanism unknown^b^	234/939 (24.9)	10/163 (6.1)	16/90 (17.8)	260/1192 (21.8)
Other	7/939 (0.8)	0	1/90 (1.1)	8/1192 (0.7)
Imaging findings				
Skull fracture	0	17/173 (9.8)	5/98 (5.1)	22/1264 (1.7)
Epidural or extradural hematoma	0	3/173 (1.7)	3/98 (3.1)	6/1264 (0.5)
Subdural hematoma	0	11/173 (6.4)	3/98 (3.1)	14/1264 (1.1)
Subarachnoid hemorrhage	0	32/173 (18.5)	20/98 (20.4)	52/1264 (4.1)
Intraparenchymal hemorrhage	0	17/173 (9.8)	15/98 (15.3)	32/1264 (2.5)
Intraventricular hemorrhage	0	3/173 (1.7)	7/98 (7.1)	10/1264 (0.8)
Cerebral edema	0	1/173 (0.6)	5/98 (5.1)	6/1264 (0.5)
Diffuse axonal injury	0	1/173 (0.6)	0	1/1264 (0.1)
Midline shift	0	14/173 (8.1)	13/98 (13.3)	27/1264 (2.1)
Highest level of care				
Hospital^c^	722/993 (77.7)	153/173 (88.4)	78/98 (79.6)	1003/1264 (79.4)
Outpatient	68/993 (6.9)	5/173 (2.9)	1/98 (1.0)	74/1264 (5.9)
Evaluation sought but level unclear	108/993 (10.9)	4/173 (2.3)	2/98 (2.0)	114/1264 (9.0)
**Offspring cohort**				
TBIs	1474/1902 (77.5)	266/1902 (14.0)	162/1902 (8.5)	1902/1902 (100)
Sex				
Female	806/1474 (54.7)	145/266 (54.5)	80/162 (49.4)	1031/1902 (54.2)
Male	668/1474 (45.3)	121/266 (45.5)	82/162 (50.6)	871/1902 (45.8)
Age at injury, mean (SD) [range], y	70 (15) [11-101]	72 (14) [30-92]	70 (17) [17-99]	71 (15) [11-101]
Setting of injury				
Residential	683/1136 (60.1)	141/230 (61.3)	89/141 (63.1)	913/1507 (60.6)
Recreational	191/136 (16.8)	42/230 (18.3)	15/141 (10.6)	248/1507 (16.5)
Transportation related^b^	181/1136 (15.9)	27/230 (11.7)	23/141 (16.3)	231/1507 (15.3)
Occupational	26/1136 (2.3)	3/230 (1.3)	8/141 (5.7)	37/1507 (2.5)
Organized sports	24/1136 (2.1)	1/230 (0.4)	0	25/1507 (1.7)
Military	1/1136 (0.1)	4/230 (1.7)	0	5/1507 (0.3)
Other	30/1136 (2.6)	12/230 (5.2)	6/141 (4.3)	48/1507 (3.2)
Mechanism of injury				
Acceleration or deceleration	50/1301 (3.8)	3/244 (1.2)	2/154 (1.3)	55/1699 (3.2)
Head struck by or against an object	161/1301 (12.4)	36/244 (14.8)	25/154 (16.2)	222/1699 (13.1)
Fall with trauma to the head	1088/1301 (83.6)	200/244 (82.0)	119/154 (77.3)	1407/1699 (82.8)
Force generated by a blast or explosion	1/1301 (0.1)	0	0	1/1699 (0.1)
Foreign body penetrating the brain	0	2/244 (0.8)	8/154 (5.2)	10/1699 (0.6)
Other	1/1301 (0.1)	3/244 (1.2)	0	4/1699 (0.2)
Imaging findings				
Skull fracture	0	14/266 (5.3)	9/162 (5.6)	23/1902 (1.2)
Epidural or extradural hematoma	0	11/266 (4.1)	4/162 (2.5)	15/1902 (0.8)
Subdural hematoma	0	74/266 (27.8)	55/162 (34.0)	129/1902 (6.8)
Subarachnoid hemorrhage	0	75/266 (28.2)	17/162 (10.5)	92/1902 (4.8)
Intraparenchymal hemorrhage	0	22/266 (8.3)	26/162 (16.0)	48/1902 (2.5)
Intraventricular hemorrhage	0	10/266 (3.8)	6/162 (3.7)	16/1902 (0.8)
Cerebral edema	0	5/266 (1.9)	11/162 (6.8)	16/1902 (0.8)
Diffuse axonal injury	0	3/266 (1.1)	0	3/1902 (0.2)
Midline shift	0	24/266 (9.0)	25/162 (15.4)	49/1902 (2.6)
Highest level of care				
Outpatient	113/1474 (7.7)	11/266 (4.1)	15/162 (9.3)	139/1902 (7.3)
Emergency department	714/1474 (48.4)	78/266 (29.3)	48/162 (29.6)	840/1902 (44.2)
Inpatient, not ICU	353/1474 (24.0)	105/266 (39.5)	50/162 (30.9)	508/1902 (26.7)
ICU	17/1474 (1.2)	26/266 (9.8)	22/162 (13.6)	65/1902 (3.4)
Evaluation sought but level unclear	63/1474 (4.3)	7/266 (2.6)	3/162 (1.9)	73/1902 (3.8)

Abbreviations: ICU, intensive care unit; TBI, traumatic brain injury.

^a^ Unless indicated otherwise, values are presented as No./total No. (%) of TBIs.

^b^ The mechanism “motor vehicle crash, mechanism unknown” was excluded in the offspring cohort. Motor vehicle crashes were instead denoted by “transportation related” as setting of injury. More granular categories for highest level of care were used in the offspring cohort.

^c^ Including emergency department evaluation, without admission.

Incidence rates were higher in the offspring cohort compared with the original cohort. Among the original cohort, incidence was 7.02 (95% CI, 6.63-7.40) TBI events per 1000 person-years. By severity strata, incidence (in TBI events per 1000 person-years) in the original cohort was 6.06 (95% CI, 5.70-6.42) for mild TBI, 0.41 (95% CI, 0.32-0.50) for moderate TBI, and 0.54 (95% CI, 0.44-0.65) for severe TBI. Female participants experienced TBIs 26.9% more frequently (7.68 [95% CI, 7.16-8.21] TBI events per 1000 person-years) compared with male participants (6.05 [95% CI, 5.49-6.61] TBI events per 1000 person-years).

Among the offspring cohort, incidence was 9.11 (95% CI, 8.70-9.51) TBI events per 1000 person-years, which was 29.8% higher compared with 7.02 in the original cohort. By severity strata, incidence (in TBI events per 1000 person-years) in the offspring cohort was 7.06 (95% CI, 6.70-7.42) for mild TBI, 0.46 (95% CI, 0.37-0.56) for moderate TBI, and 1.58 (95% CI, 1.41-1.76) for severe TBI. Males experienced 9.06 (95% CI, 8.46-9.67) TBI events per 1000 person-years, and females experienced 9.14 (95% CI, 8.58-9.70) TBI events per 1000 person-years.

**Figure 1.**
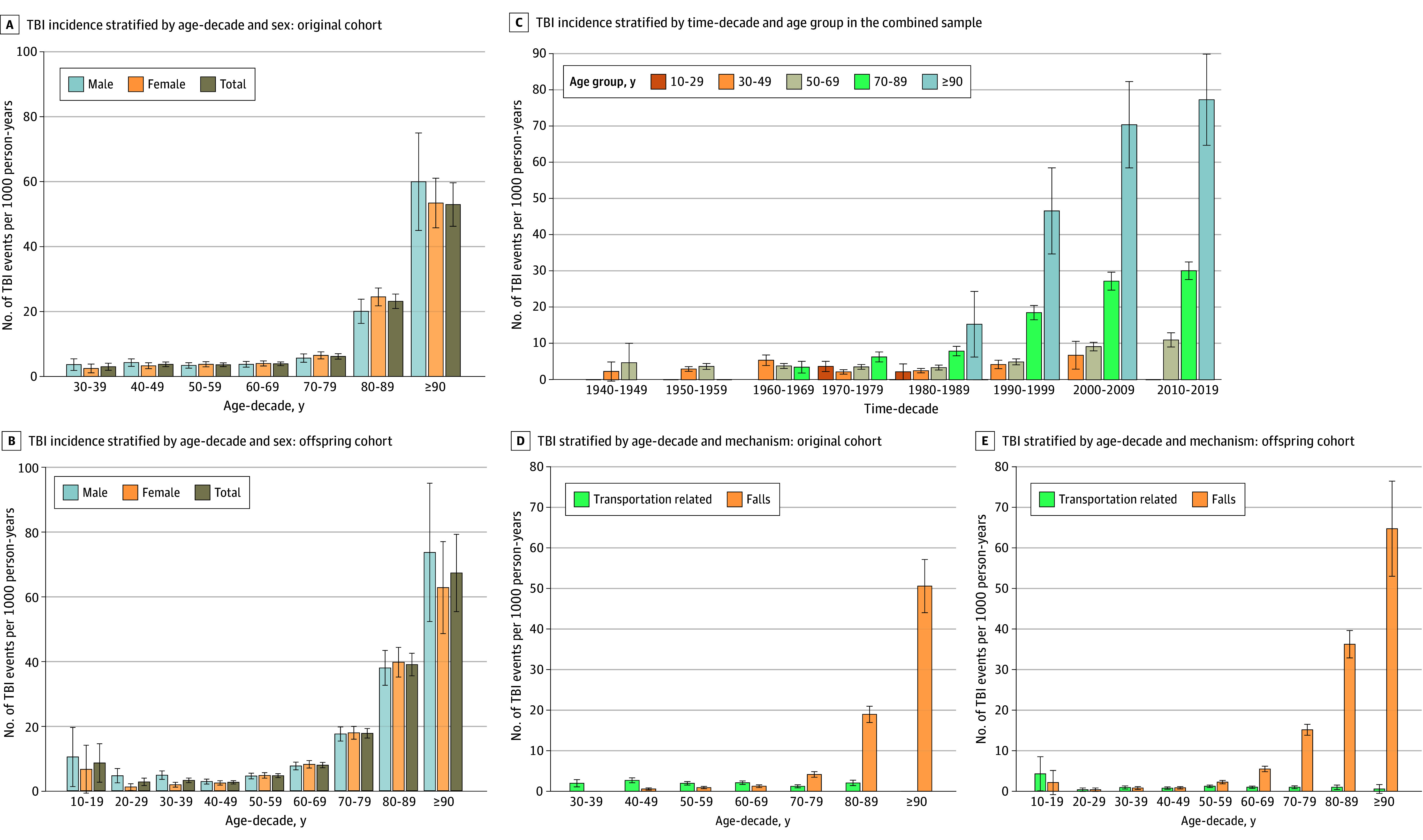
Incidence of Traumatic Brain Injury (TBI) in the Framingham Heart Study Original and Offspring Cohorts A and B, TBI incidence stratified by age-decade and sex among the original (A) and offspring (B) cohorts. C, TBI incidence stratified by time-decade and age group in the combined sample. D and E, TBI incidence stratified by age-decade and mechanism of TBI (transportation-related, falls) among the original (D) and offspring (E) cohorts. TBI incidence is reported in number of TBI events per 1000 person-years.

Figure 1A and B show TBI incidence stratified by age-decade and sex for the original and offspring cohorts, respectively. In both cohorts, TBI incidence increased markedly with older age (particularly ages 70-89 and ≥90 years), with rates growing rapidly in the 8th, 9th, and 10th decades of life. Figure 1C illustrates TBI incidence in both cohorts, stratified by time-decade and age. Within the same age group, TBI incidence increased beginning in the 1990s, with greater increases in the 2000s and 2010s. This increase was most pronounced among the older age groups. Figure 1D and E illustrate TBI incidence from transportation-related mechanisms (eg, motor vehicle crashes) and falls for the original and offspring cohorts, respectively. Among the original cohort, the most common TBI mechanisms were transportation related (72.1%) for those aged younger than 70 years and falls (83.8%) for those aged 70 years or older. Among the offspring cohort, transportation-related mechanisms (32.7%) and falls (32.1%) had similar rates for those aged younger than 50 years, and falls (81.5%) were the most common mechanism for those aged 50 years or older.

**Figure 2.**
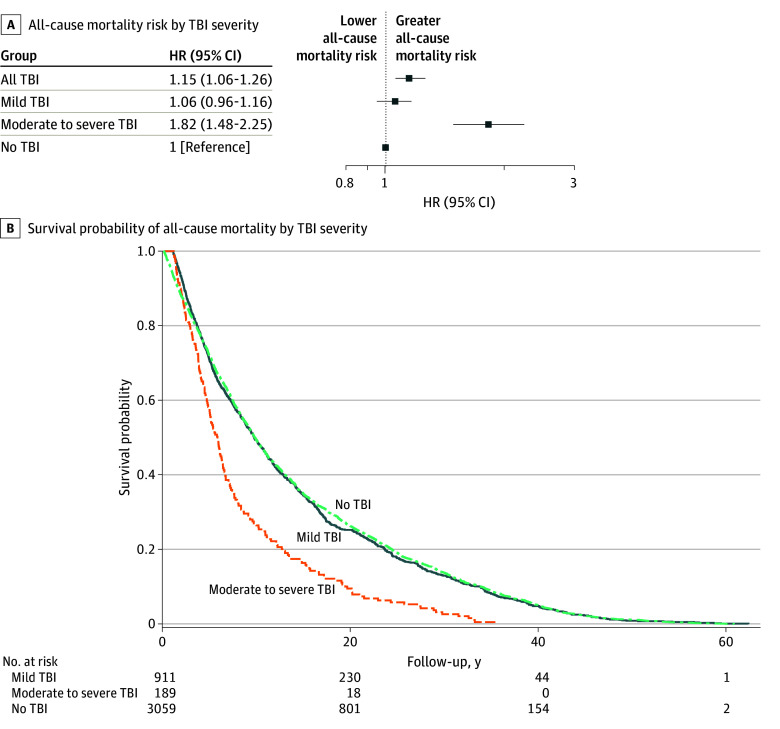
Adjusted Hazard Ratios (HRs) for the Association Between Traumatic Brain Injury (TBI) and All-Cause Mortality A, Forest plot of all-cause mortality risk by TBI severity. B, Survival probability by TBI severity. Participants (n = 6752) were matched between those with and without TBI in a 1:3 ratio. Follow-up served as the time scale. All models were adjusted for covariates including education status, cigarette smoking, diabetes, hypertension, cardiovascular disease, and stroke history.

eFigure 2 in Supplement 1 illustrates the flow of included participants in the time-to-event analyses. Baseline characteristics by TBI status within the matched sample are presented in eTable 2 in Supplement 2. Figure 2 shows the hazard ratios (HRs) and survival probabilities of all-cause mortality for TBI occurrence and severity. TBI was associated with all-cause mortality, with an HR of 1.15 (95% CI, 1.06-1.26) among participants with TBI compared with those without TBI. Moderate to severe TBI, but not mild TBI, was associated with all-cause mortality. The HRs for all-cause mortality among participants with mild and moderate to severe TBI were 1.06 (95% CI, 0.96-1.16) and 1.82 (95% CI, 1.48-2.25), respectively, compared with those without TBI. The Kaplan-Meier curve plots for all-cause mortality showed a more rapid decrease in cumulative survival among participants with moderate to severe TBI compared with those without TBI (Figure 2). The decrease in cumulative survival was similar for participants with mild TBI and those without TBI. Given the observed differences between mild and moderate to severe TBI, further results are presented stratified by TBI severity.

**Figure 3.**
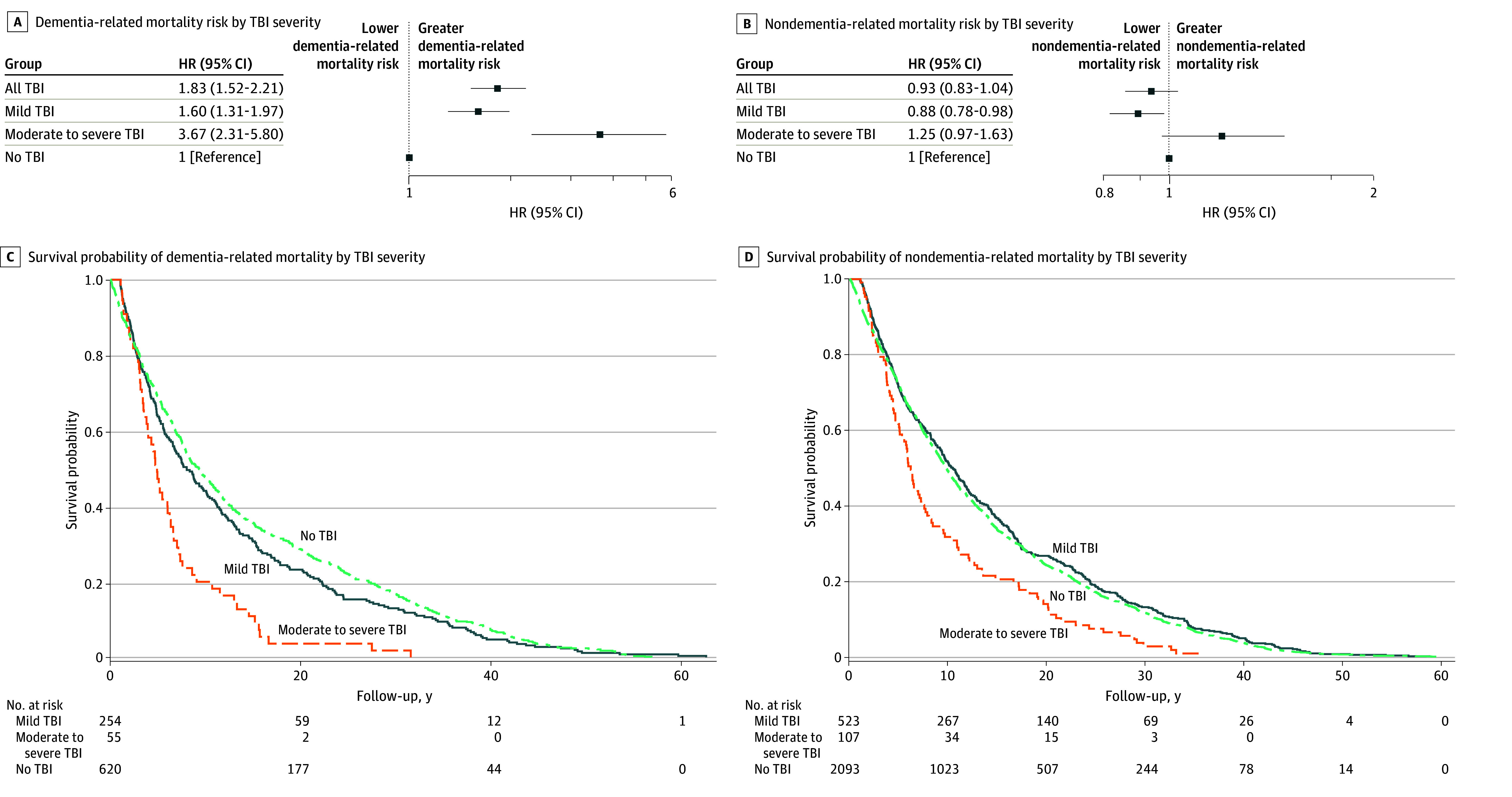
Adjusted Hazards Ratio (HR) for the Association Between Traumatic Brain Injury (TBI) and Dementia-Related and Non–Dementia-Related Mortality A and B, Dementia-related (A) and non–dementia-related (B) mortality risk by TBI severity. C and D, Survival probability of dementia-related (C) and non–dementia-related (D) mortality by TBI severity. Participants (n = 6752) were matched between those with and without TBI in a 1:3 ratio. Follow-up served as the time scale. All models were adjusted for covariates including education status, cigarette smoking, diabetes, hypertension, cardiovascular disease, and stroke history.

Dementia-related and non–dementia-related mortality results are illustrated in Figure 3. Mild TBI and moderate to severe TBI were associated with dementia-related mortality. The HRs for dementia-related mortality among participants with mild and moderate to severe TBI were 1.60 (95% CI, 1.31-1.97) and 3.67 (95% CI, 2.31-5.80), respectively, compared with those without TBI. The Kaplan-Meier curve plots in Figure 3C and D for dementia-related mortality show a more rapid decrease in cumulative survival among participants with mild and moderate to severe TBI compared with those without TBI. HRs for non–dementia-related mortality among participants with mild and moderate to severe TBI were not significantly different compared with those without TBI, and Kaplan-Meier curve plots showed similar decreases across groups. The proportional hazards assumption was satisfied for all models.

In sensitivity analyses among individuals with TBI prior to cognitive impairment onset and matched control participants (without cognitive impairment and TBI), TBI remained associated with both time to all-cause mortality and dementia-related mortality, with similar association sizes as those observed in the primary analyses (eTable 10 in Supplement 1). In sensitivity analyses among participants with cognitive impairment, TBI after cognitive impairment onset was not associated with time to all-cause or dementia-related mortality (eTable 10 in Supplement 1). In sensitivity analyses excluding participants who died within 2 years of TBI and matched control participants, association sizes remained similar to the primary analyses (eTable 11 in Supplement 1). In secondary analyses, we did not observe significant TBI × sex or TBI × age strata interactions on all-cause mortality or dementia-related mortality. Stratified analyses by sex (eTables 3 and 4 in Supplement 1) showed nonsignificantly larger association magnitudes for men for mild TBI and for women for moderate to severe TBI for both all-cause and dementia-related mortality. Stratified analyses by age (eTables 5 and 6 in Supplement 1) showed nonsignificantly larger associations for mild TBI for age 60 years or older and for moderate to severe TBI for age younger than 60 years for both all-cause and dementia-related mortality. Among participants with TBI, HRs for all-cause mortality and dementia-related mortality for multiple TBIs compared with single TBI were 2.00 (95% CI, 1.66-2.41) and 3.61 (95% CI, 2.42-5.39), respectively (eTables 7 and 8 in Supplement 1). In models that examined categories of non–dementia-related mortality, there was an association between moderate to severe TBI and CVA-related mortality, although the 95% CI was very large (HR, 18.46 [95% CI, 2.21-154.34]) (eTable 9 in Supplement 1). In models that examined categories of dementia-related mortality, association size was modestly larger for other dementia-related mortality than pure AD dementia-related mortality (eTable 12 in Supplement 1).

## Discussion

This longitudinal cohort study used medical and self-report data collected through comprehensive review of the 10 333 total participants in the FHS original and offspring cohorts to investigate the lifetime incidence of TBI and its effect on long-term all-cause and dementia-related mortality. We found the lifetime incidence of TBI to be 7.02 TBI events per 1000 person-years in the original cohort and 9.11 TBI events per 1000 person-years in the offspring cohort. TBI incidence increased with increasing age- and time-decades, with the most common mechanisms of injury being transportation-related events and falls. TBI was associated with all-cause mortality, with the association largely attributable to dementia-related mortality. We observed a dose-response association for mild vs moderate to severe TBI and for having a single TBI vs multiple TBIs.

Marked differences in TBI incidence were present between the FHS original and offspring cohorts. Notably, rates of TBI were 30.0% higher among the offspring cohort, with this outcome most pronounced at older ages. External factors, including increasing public awareness of TBI, improving documentation in electronic medical record systems, and shifting emphasis toward increased reporting following the Centers for Disease Control and Prevention initiative for TBI surveillance in 1989, may have contributed.^2,40,41^ Using composite data from both the original and offspring cohorts, we observed that TBI incidence increased across time-decades, even when stratified by age group. This outcome was most notable in older age groups (aged 70-89 and ≥90 years), particularly from the 1990s onward. This finding suggests that although an aging cohort did contribute to increased TBI incidence, external factors such as public awareness, increased reporting, and improved documentation also contributed.^2^

Additionally, we observed cohort-specific differences in the mechanisms of TBI. In the FHS original cohort, motor vehicle crashes were the most common mechanism of TBI among younger participants (aged <70 years), whereas TBIs due to falls were most common among older participants (aged ≥70 years). Interestingly, in the FHS offspring cohort, TBIs due to motor vehicle crashes and falls were similarly common in participants aged younger than 50 years, whereas TBIs due to falls were most common among participants aged 50 years or older. These cohort-specific differences are likely the by-product of seat belt usage, which increased substantially following the adoption of mandated seat belt laws in the mid-1980s.^41,42^ Consistent with previous literature, there was a substantial increase in TBIs due to falls with each subsequent age-decade in both cohorts in the present study, highlighting the substantial medical burden that falls represent with aging.^7,8^ The TBI incidence rate differed between sexes in the original cohort, with female participants experiencing TBIs 27.0% more frequently. However, this outcome was not observed when TBI incidence was stratified by age-decade, likely because female participants lived to older ages, leading to higher representation in the older age groups where late-life TBI and dementia are more prevalent.

TBI was associated with all-cause mortality in time-to-event analyses. This result is consistent with studies of mortality and TBI identified in hospital and rehabilitation settings as well as in the community.^14,43,44,45^ Additionally, TBI (both mild and moderate to severe) was associated with dementia-related mortality, but not with non–dementia-related mortality, in time-to-event analyses. These findings build on a 2023 community-based study showing that neurologic causes of death, particularly from neurodegenerative diseases, were more common in participants with TBI compared with those without TBI.^14^ TBI is a well-known modifiable risk factor for dementia, although the circumstances for which this association is greatest (eg, age at TBI or sex) require further investigation.^46,47,48,49^ Consistently, our findings suggest an association among TBI, dementia, and mortality.^50^ Future research leveraging longitudinal data that carefully disentangle the causal direction of these associations is needed.

A dose-response association for mild TBI vs moderate to severe TBI and for single TBI vs multiple TBIs was observed for all-cause mortality, providing added confidence in these observed associations. Similar dose-response patterns have been previously observed both in hospital and community-based settings.^14,44,51^ In this study, these dose-response associations extended to dementia-related mortality, which appeared to account for much of the association. Collectively, these results support the hypothesis that a history of TBI functions as a chronic condition contributing to both neurodegenerative disease and overall mortality. These findings underscore the critical need to prevent TBI, particularly from falls, to reduce its enduring consequences, particularly concerning cognitive health and dementia risk.

### Strengths and Limitations

The strengths of our study include the representative, community-based sample, enrollment of many participants at a young age, careful longitudinal follow-up with low attrition across 7 decades, and comprehensive surveillance of TBI by self-report and rigorous medical record review, including records from hospital visits, outpatient and primary care visits, nursing home and hospice records, and death certificates. Our study also has limitations. The FHS original and offspring cohorts were mainly composed of well-educated, self-reported non-Hispanic White participants, thus limiting generalizability.^52^ Due to FHS study health surveillance, participants tended to be healthier than nonparticipants, and this could have resulted in underestimation of TBI incidence and mortality risk. TBI surveillance via medical record review spanned the 1940s until 2021. Although this offered a unique window into the changing landscape of TBI diagnosis and care over time, it also introduced variation in TBI documentation due to the increased awareness and surveillance of TBI that occurred during the late 1900s.

Study data collection was usually limited to medical information collected after participant enrollment, which limited ascertainment of TBIs during childhood and young adulthood. The self-report questionnaire on TBI was limited in scope, changed over time, and was not administered at every study visit. We recently began collecting more comprehensive self-report data using the Ohio State University TBI Identification Method,^53^ with additional questions on repetitive head impacts from contact sports, military service, and physical violence, but data collection is still underway.

When choosing matches without TBI, we did not allow for the matches to have a future TBI because future TBI may mediate the association between an earlier TBI and mortality, potentially biasing estimation. Nonetheless, their exclusion as potential matches could also introduce bias, more likely toward the null.

Dementia-related deaths were defined using adjudicated dementia diagnoses and the FHS’s comprehensive death review. Although dementia-related cause of death was not one of the coded categories for clinicians to choose in these death reviews, our gold-standard adjudication of dementia together with other causes (ie, that were not CVD, CVA, cancer, other causes [without having been adjudicated to have dementia], or unknown causes) provides a reasonable approximation of dementia-related death that other population-based studies of TBI and mortality do not have. Nonetheless, we recognize that in some cases we may have misattributed the cause of death to dementia. Accounting in our models for additional potential confounders, including the apolipoprotein *APOE* ε4 allele, physical activity, alcohol use, frailty, and depression, may have been useful, but we were unable to include them due to substantial missing data or because they were measured after the TBI.

### Conclusions

This longitudinal cohort study documented TBI incidence over 7 decades, showing increasing incidence with age and over time, with the majority of TBIs related to falls. TBI was associated with an increased risk of all-cause mortality, with the association largely attributable to dementia-related mortality. There was a dose-response association for the number and severity of TBIs. These findings provide added support for TBI to be considered a chronic condition, and they suggest that preventing falls to reduce TBI could have important implications for dementia and mortality.

## Notes

Supplement 1.
eMethods.
**eFigure 1.** Data Cleaning Workflow **eFigure 2.** Participant Selection Flow Chart for Time-to-Event Analyses Among the Nested Matched Samples in the Original and Offspring Cohorts **eTable 1.** Modified American Congress of Rehabilitation Medicine and US Department of Veterans Affairs and Department of Defense Guidelines for Traumatic Brain Injury (TBI) Classification **eTable 2.** Demographics by Matched Sample (n = 6752) **eTable 3.** Adjusted Hazard Ratios (HRs) for the Association Between Traumatic Brain Injury (TBI) and All-Cause Mortality by Sex **eTable 4.** Adjusted Hazard Ratios (HRs) for the Association Between Traumatic Brain Injury (TBI) and Dementia-Related Mortality by Sex **eTable 5.** Adjusted Hazard Ratios (HRs) for the Association Between Traumatic Brain Injury (TBI) and All-Cause Mortality by Age at TBI (<60 and ≥60 Years) **eTable 6.** Adjusted Hazard Ratios (HRs) for the Association Between Traumatic Brain Injury (TBI) and Dementia-Related Mortality by Age at TBI (<60 and ≥60 Years) **eTable 7.** Adjusted Hazard Ratios (HRs) for the Association Between Traumatic Brain Injury (TBI) and All-Cause Mortality by TBI Count (n = 1617) **eTable 8.** Adjusted Hazard Ratios (HRs) for the Association Between Traumatic Brain Injury (TBI) and Dementia-Related Mortality by TBI Count (n = 1617) **eTable 9.** Adjusted Hazard Ratios (HRs) for the Association Between Traumatic Brain Injury (TBI) and Cause-Specific Mortality (n = 6752) **eTable 10.** Sensitivity Analysis Exploring Reverse Causality **eTable 11.** Sensitivity Analysis Using a 2-Year Threshold Between Traumatic Brain Injury (TBI) and Death **eTable 12.** Sensitivity Analysis Stratifying Dementia-Related Mortality Into Pure Alzheimer Disease (AD)–Related Mortality and Other Dementia-Related Mortality (n = 6752)
eReferences.

Supplement 2.
Data Sharing Statement
