# Supplementary material for: Traumatic Brain Injury and All-Cause and Dementia-Related Mortality in the Framingham Heart Study
Source: JAMA Netw Open. 2026 Jan 30;9(1):e2555138. doi: 10.1001/jamanetworkopen.2025.55138 (PMC12859724; doi:10.1001/jamanetworkopen.2025.55138)
Supplement: Supplement 1. — eMethods. eFigure 1. Data Cleaning Workflow eFigure 2. Participant Selection Flow Chart for Time-to-Event Analyses Among the Nested Matched Samples in the Original and Offspring Cohorts eTable 1. Modified American Congress of Rehabilitation Medicine and US Department of Veterans Affairs and Department of Defense Guidelines for Traumatic Brain Injury (TBI) Classification eTable 2. Demographics by Matched Sample (n = 6752) eTable 3. Adjusted Hazard Ratios (HRs) for the Association Between Traumatic Brain Injury (TBI) and All-Cause Mortality by Sex eTable 4. Adjusted Hazard Ratios (HRs) for the Association Between Traumatic Brain Injury (TBI) and Dementia-Related Mortality by Sex eTable 5. Adjusted Hazard Ratios (HRs) for the Association Between Traumatic Brain Injury (TBI) and All-Cause Mortality by Age at TBI (<60 and ≥60 Years) eTable 6. Adjusted Hazard Ratios (HRs) for the Association Between Traumatic Brain Injury (TBI) and Dementia-Related Mortality by Age at TBI (<60 and ≥60 Years) eTable 7. Adjusted Hazard Ratios (HRs) for the Association Between Traumatic Brain Injury (TBI) and All-Cause Mortality by TBI Count (n = 1617) eTable 8. Adjusted Hazard Ratios (HRs) for the Association Between Traumatic Brain Injury (TBI) and Dementia-Related Mortality by TBI Count (n = 1617) eTable 9. Adjusted Hazard Ratios (HRs) for the Association Between Traumatic Brain Injury (TBI) and Cause-Specific Mortality (n = 6752) eTable 10. Sensitivity Analysis Exploring Reverse Causality eTable 11. Sensitivity Analysis Using a 2-Year Threshold Between Traumatic Brain Injury (TBI) and Death eTable 12. Sensitivity Analysis Stratifying Dementia-Related Mortality Into Pure Alzheimer Disease (AD)–Related Mortality and Other Dementia-Related Mortality (n = 6752) eReferences. [file jamanetwopen-e2555138-s001.pdf]

Supplemental Online Content

Burton R, Durape S, Price E, et al. Traumatic brain injury and all-cause and dementia-related mortality in the Framingham Heart Study. *JAMA Netw Open*. 2026;9(1):e2555138. doi:10.1001/jamanetworkopen.2025.55138

eMethods.

eFigure 1. Data Cleaning Workflow

eFigure 2. Participant Selection Flow Chart for Time-to-Event Analyses Among the Nested Matched Samples in the Original and Offspring Cohorts

eTable 1. Modified American Congress of Rehabilitation Medicine and US Department of Veterans Affairs and Department of Defense Guidelines for Traumatic Brain Injury (TBI) Classification

eTable 2. Demographics by Matched Sample (n = 6752)

eTable 3. Adjusted Hazard Ratios (HRs) for the Association Between Traumatic Brain Injury (TBI) and All-Cause Mortality by Sex

eTable 4. Adjusted Hazard Ratios (HRs) for the Association Between Traumatic Brain Injury (TBI) and Dementia-Related Mortality by Sex

eTable 5. Adjusted Hazard Ratios (HRs) for the Association Between Traumatic Brain Injury (TBI) and All-Cause Mortality by Age at TBI (<60 and ≥60 Years)

eTable 6. Adjusted Hazard Ratios (HRs) for the Association Between Traumatic Brain Injury (TBI) and Dementia-Related Mortality by Age at TBI (<60 and ≥60 Years)

eTable 7. Adjusted Hazard Ratios (HRs) for the Association Between Traumatic Brain Injury (TBI) and All-Cause Mortality by TBI Count (n = 1617)

eTable 8. Adjusted Hazard Ratios (HRs) for the Association Between Traumatic Brain Injury (TBI) and Dementia-Related Mortality by TBI Count (n = 1617)

eTable 9. Adjusted Hazard Ratios (HRs) for the Association Between Traumatic Brain Injury (TBI) and Cause-Specific Mortality (n = 6752)

eTable 10. Sensitivity Analysis Exploring Reverse Causality

eTable 11. Sensitivity Analysis Using a 2-Year Threshold Between Traumatic Brain Injury (TBI) and Death

eTable 12. Sensitivity Analysis Stratifying Dementia-Related Mortality Into Pure Alzheimer Disease (AD)–Related Mortality and Other Dementia-Related Mortality (n = 6752)

eReferences.

This supplemental material has been provided by the authors to give readers additional information about their work.

© 2026 Burton R et al. *JAMA Network Open*.

eMethods.

FHS Recruitment and Consent

FHS participants for the Original Cohort were recruited through community outreach and canvassing throughout Framingham, Massachusetts starting in 1948. FHS personnel sent recruitment letters to a random sampling of two of every three families with household members within the age range of 30-59 years. The Offspring Cohort, as implied through its name, is composed of the children of the Original Cohort and their spouses and were recruited through outreach through the Original Cohort participants' families. Data collection for both cohorts began when the participants were first enrolled in the study. Written consent for the main FHS exam and ancillary studies (such as this one) were obtained by participants at initial enrollment, during each exam cycle, and at the start of participation in ancillary studies. For more information on FHS participant recruitment, cohort information, and exam visits and participant tracking, see Dawber et al. (1951), Feinleib et al. (1975), and Satizabal et al. (2016). The institutional review board at the Boston University Medical Center approved study protocols and consent forms for and maintained supervision of FHS and this study.

FHS Participant Data Collection

Each enrolled FHS participant has a "chart" that is compiled of folders for each exam cycle with participant information, FHS exams reports, and medical documentation. The Original Cohort underwent exams every two years, and Offspring Cohort exams have occurred every four years and are still ongoing.[3] Participant information includes demographic information and was updated each exam visit to ensure demographic and socioeconomic information (*e.g.*, education, occupation, marital status, etc.) remains accurate. On top of demographic information, a comprehensive study exam was conducted to collect a wide range of health information (*e.g.*, medical history since last visit, medications, cardiovascular health, bone health, lung health, neurocognitive battery, etc.).[4] All participants were invited for neurological and cognitive assessments as ancillary visits, with added invitations if a participant was flagged for potential cognitive or neurological issues by FHS personnel, family members, or physicians. In between exam cycles/examinations, participants and/or informants completed questionnaires detailing medical and family history updates to maintain surveillance.[4] Participant medical records and findings were released from hospitals, nursing facilities, urgent care, and outpatient visits from the time of FHS recruitment until death. Radiology images themselves were not reviewed for this study, but radiology reports and findings were reviewed as they were included in the participants' charts. Participants were also asked head injury related questions at each exam. See below for details on how this question was asked for each cohort. For more information regarding the data FHS exam visits collect, see Tsao & Ramachandran (2015).

**Original Cohort**	<u>Exam 19</u>: Ever had a head injury causing loss of consciousness?<br><u>Exam 22</u>: Did any of your falls in the past year result in a head injury requiring medical attention?<br><u>Exam 23</u>: Did any of your falls in the past year result in a head injury requiring medical attention?

© 2026 Burton R et al. *JAMA Network Open*.

**Offspring Cohort**	Exam 24: Did any of your falls in the past year result in a head injury requiring medical attention?<br>Exam 7: History of ever having a head injury with loss of consciousness?<br>Exam 8: History (since your last exam) of having a head injury with loss of consciousness?<br>Exam 9: History (since your last exam) of having a head injury with loss of consciousness?

Chart Review Training

The chart review for this study was conducted by extensively trained study personnel between the years of 2019 and 2022. These study personnel were senior and junior research assistants (RAs). Chart review training procedures for both levels of research assistants began with training on FHS history and current studies, chart room training (pulling and storing participant charts), TBI definition and classification, data confidentiality and collection, and TBI Project procedural overview. Following two days of orientation, both levels of research assistants began the extensive chart review training process. Training procedures involved reviewing "training" charts, which were charts that had been previously reviewed and quality control checked (QC'ed) with an answer key generated to compare trainees' answers to. Trainees (with supervision) pulled participants' charts, independently reviewed the chart and entered the data into a separate REDCap training project and received individual feedback on any deviations from the answer key. Junior RA trainees reviewed a minimum of ten training charts of varying difficulty levels (assigned and monitored by senior RAs) but received more training charts if necessary to reach required accuracy levels. On average, junior RAs reviewed between 10 and 15 training charts before moving to official chart review. Junior and senior RA training processes were similar, but senior RAs had to complete a higher number of training charts (*i.e.*, 30+ training charts) since they needed to be experienced enough to oversee junior RA chart review. After training, trainees moved into a strict QC review process where 20% of charts were reviewed and where the QC occurred sooner than the regular QC process. For junior RAs, the initial QC reviewed one out of every five participants, and the official QC pulled four participants of each set of 20 participants reviewed. For senior RAs, the initial strict QC pulled four out of a batch of 20 participants, and the official QC pulled five out of every 100 participants reviewed. If the strict QC process proved satisfactory, personnel would move on to official chart review with regular QC; otherwise, they would remain at the strict QC level for another round. If that second round of strict QC did not match the required accuracy level, those personnel returned to training. Dr. Jesse Mez (Principal Investigator) and the data management team responsible for QC reports decided when junior and senior research assistants were cleared from training and initial strict QC.

Chart Review Data Collection

To begin the process of data collection, data managers for the TBI Project randomly assigned participants for each study personnel to review. Study personnel then reviewed the entirety of a participant's chart, which ranged between 500 to 1,000 pages in total per participant. Each page in the Original Cohort's charts were digitized and accessed by personnel via FHS's secure digital storage platform. The charts for

© 2026 Burton R et al. *JAMA Network Open*.

the Offspring Cohort were paper charts that were reviewed by hand by personnel. As study personnel went through each participant's chart, they looked for confounding factors and any instance(s) of head injury and recorded data via REDCap electronic data capture tools.[5] Potential confounding factor information was also collected for each participant, regardless of head injury status. Potential confounding factors were pre-existing (prior to head trauma(s)) conditions/issues that could potentially (immediately/directly) lead to/cause a head injury to occur an/or may be impacting clinical manifestations (head injury or post-injury symptoms) of the head injury. Potential confounding factors included recreational drug use; treatment related complications; psychological trauma from an event; schizophrenia; depressive disorders; bipolar disorders; learning disabilities; general anxiety disorder; post-traumatic stress disorder; organ failure; and other confounding factors. It should be noted that if these confounding factors made it challenging for study personnel to accurately record head injury data, then personnel were instructed to "flag" the case for more senior personnel to review. The flagged case process is detailed later in the Supplement Methods.

If there was no evidence of head injury in the chart, personnel recorded "No head injury" in REDCap for the participant and listed any potential confounding factors. If study personnel found one or more instances of head injury, personnel recorded all available head injury data into REDCap. If there were multiple head injuries over a participant's lifetime, personnel filled out a head injury record form for the first head injury, and then continued adding new head injury records. Personnel could enter up to a maximum of 5 head injuries and were instructed to report the first head injury followed by the four most severe head injuries. Our study's "head injury record" in REDCap began with documentation of date and source of information. Personnel first recorded the date of the injury, the exam cycle the head injury occurred in, and the informational source(s) available for review for the head injury (*i.e.*, medical records, FHS exam records, other). If personnel selected medical records, they would then record what kind of medical record was being used for data collection and the date of each medical record. If head injury information was reported in both medical records and FHS exam records with discrepant information, personnel were instructed that medical records would take precedence over self-reported information in the FHS exam records. Time between injury and presentation to care and highest level of care for the injury were also recorded. Personnel then recorded information regarding the setting (*i.e.*, residential, recreational, transportational, occupational, organized sports, military, unknown, other) and mechanism (*i.e.*, acceleration/deceleration, head struck by/against an object, falls with trauma to the head; force generated by blast/explosion, foreign body penetrating the brain, unknown, other) of the head injury. If a Glasgow Coma Score (GCS) was reported in medical records, personnel documented the GCS score and how long after the head injury it was calculated. Personnel also documented whether the participant died as a result of their head injury.

The next section of the head injury REDCap form prompted personnel to record information regarding post-injury clinical symptoms (*i.e.*, loss of consciousness, post-traumatic amnesia, altered mental state, focal neurological deficits, seizures). Cerebral imaging and radiological evidence were also recorded at this point. While the actual image produced by radiology was not provided in a participant's chart, the diagnostic imaging report and/or physician/hospital conclusions were. For each imaging report, personnel documented the date of the imaging report, the type of imaging (i.e., Head CT, Head X-ray, Brain MRI, Other), chronic findings, and acute findings. Acute findings included skull fracture, ischemic stroke, epidural/extradural hematoma, subdural hematoma, subarachnoid hemorrhage, intraparenchymal

© 2026 Burton R et al. *JAMA Network Open*.

hemorrhage, intraventricular hemorrhage, cerebral contusion, cerebral edema, diffuse axonal injury, and midline shift. Our REDCap survey allowed for five imaging reports to be included. If a participant had more than five scans done at the time of the injury, personnel chose the five scans with the most relevant information on the severity of the head injury.

If available, “post-injury records” were also recorded for each head injury and were defined as follow-up/post-injury care sought for a head injury. Relevant records were considered “post-injury” for up to two years after initial head trauma. For example, a participant could have visited a physician for headaches two years after a reported head trauma and this would have still been considered a post injury record for that initial trauma. If a participant sustained a head injury, did not seek immediate attention, and then four weeks later visited a physician for a sleep disturbance (or any other related symptoms), the physician's visit would have also been considered a post-injury record. Personnel were able to record up to five post-injury records for each head injury. If a participant had more than five post-injury records for a head injury, personnel were instructed to choose the first post-injury record, the last post-injury record, and the three post-injury records in between that contained the most relevant information pertaining to the severity and post-injury sequelae of the head injury.

Flagged Case Review and Resolution

Flagged case review procedures were created for instances where, in the course of the data collection process, study personnel came across cases where it was difficult to ascertain the causes, reliability, and/or post-injury sequelae of TBI. Examples of reasons for flagging could include (but are not limited to) a stroke or seizure suspected to have occurred at the time of the head injury, but it was unclear whether the stroke preceded a TBI or vice versa, or if there were multiple conflicting medical records. As a general rule, personnel were instructed to flag cases in which they could not document head injury data with certainty or accuracy. There were three levels of review involved in our flagged case process:

1. First, the case was looked at by a staff member who was on the level of a junior RA. Cases flagged at this level were then assigned to senior RAs to resolve. The easiest cases to resolve were those that were flagged due to a misreading of the chart or a lack of familiarity with the material. Senior RAs who had worked for several months could tell if the junior reviewer simply missed something that would have clarified the issue. If this was not the case, and the senior RA could not answer the question in the flagged case, then the case was moved up to the second level of review.

2. The second level of review consisted of medical trainees or physicians with more clinical experience and expertise to understand more complicated cases. These most likely involved figuring out a clinical diagnosis based on conflicting signs and symptoms or categorizing vague symptoms. This level was where most stroke cases were resolved, as a medical student was more likely to parse the language and identify whether the stroke preceded or was caused by the TBI based on the available data. If it was still difficult to deal with a case due to a great number of confounding factors or an unusual presentation, then the case was moved to the third level of review.

3. After the second level (medical students/physicians) had reviewed the flagged case and personnel still could not make a decision/resolve the flag, then the flagged case was passed up to the principal investigator of the study (Dr. Jesse Mez) along with a brief summary of the information at hand, the reason the flagged case was still an issue, and where to find relevant information in the chart for the flagged case. Dr. Mez would then make his final judgment on the issue and explain how to proceed with the final documentation for the case.

Chart Review Quality Control (QC)

To ensure validity of data, monthly internal audits (*i.e.*, "quality control (QC) reports") were conducted. These were subdivided into inter-reviewer validity and intra-reviewer validity for analysis. To assess inter-validity, a chart was re-reviewed by a different reviewer, which could be another reviewer at the same level (senior RA vs. junior RA) or a study administrator. A randomly selected list of participants that reviewers had finished since the last QC report were generated by the study's data management team. Reviewers did not have access to any previous entries and completed the second entry of this chart and submitted QC resolution entries into a separate REDCap project. A data analyst then compared the two separate entries to find discrepancies and senior RA personnel resolved discrepancies. QC percentages differed based on where RAs were in relation to training. After training procedures were completed, 20% of reviewed charts in the first one or two QC cycles went through QC for each reviewer. If the first 20% QC was satisfactory, then the following QC reports were done for 5% of reviewed charts. Intra-reviewer validity was assessed by having the same chart reviewed a second time by the same reviewer. This QC process was done far less frequently, and only with 5% of charts completed over a month prior. Again, the reviewers did not have access to any previous entries, and these QC charts were also compared to the prior entries by the data management team and senior RA personnel resolved discrepancies.

For QC discrepancy resolution of chart review, if discrepancies were discovered through either process, discrepancies were highlighted in a report generated by the data management team and the personnel involved in the cases would jointly make the final decision on accurate documentation. For junior RAs, the junior RAs must report their conclusions to the senior RA who would make the final decision on the correct documentation. Senior RA QC resolution was completed with both senior RAs involved in the QC. If an RA made multiple errors after three regular (5%) QC cycles, the study reviewer was required to receive individual re-training by more senior study personnel and return to 20% QC.

Chart Review Data Management

Data Backup

i. **Weekly data backup of limited entries:**
    a. The data management team supervising the TBI study assessed whether any data was entered into the main TBI Gen-2 Retrospective REDCap form on a weekly basis. If new data was entered, then the data management team would export the data as a .csv file and upload it to the "data dump" on the project's secure online filing system provided by Boston University. To avoid utilization of excessive disc space, only the entries created during the week would be uploaded.

ii. **All data backup every 3 months:**

a. A data backup for all the entries to date was created every three months. This data was uploaded by the data management team to the project's secure online filing system provided by Boston University.

Data Cleaning

The data cleaning workflow was implemented every six months or upon accrual of 500 new chart entries, whichever occurred earlier. Approximately 10 rounds of data cleaning (500 charts*10=5,000) were implemented before the generation of the final TBI-Gen2 dataset. To begin this process, the participant IDs were compared with the master list of FHS Gen-2 participants provided by FHS. A list of IDs that did not match the master list ("ghost IDs") were sent to the senior RA in charge of the study. Next, the charts that had been flagged were separated and cleaned when the entry was confirmed by the appropriate reviewers. The subsequent steps included checking and flagging of duplicate/multiple entries with the same participant ID and checking for inconsistencies in the branching logic in the REDCap form. The participant IDs from the charts that did not follow the above-mentioned logic flow were communicated with the research assistant. The data analyst and RA would make a joint decision regarding the duplicate entry and REDCap logic flow. Next, the analyst would map out and merge appropriate variables, convert character values to numeric values, transform the data, and check for logic steps such as dates, missing values, and post-injuries without head injuries. The cleaned dataset/table generated at the end of each data cleaning cycle was verified by another data analyst and was then merged with the previously cleaned dataset. The merged dataset was also assessed by the senior RA. See the Offspring Cohort ("Gen 2") mapped data cleaning workflow below (eFigure 1).

Traumatic Brain Injury Data Classification

Injuries were categorized into either "Head injury, but not meeting TBI criteria" or "TBI" which was further categorized based on severity into "Mild," "Moderate," or "Severe." The presence of any single criterion from the higher severity fulfilled requirements for that severity (e.g., abnormal imaging automatically elevated the injury to "Moderate" severity). Classification of TBI was modified from existing ACRM (American Congress of Rehabilitation Medicine) and the VA/DoD (U.S. Department of Veterans Affairs and Department of Defense) guidelines to include assessment of seizures, requirement of brain surgery due to TBI, and death due to TBI as shown in **eTable 1**.

© 2026 Burton R et al. *JAMA Network Open*.

eFigure 1. Data Cleaning Workflow

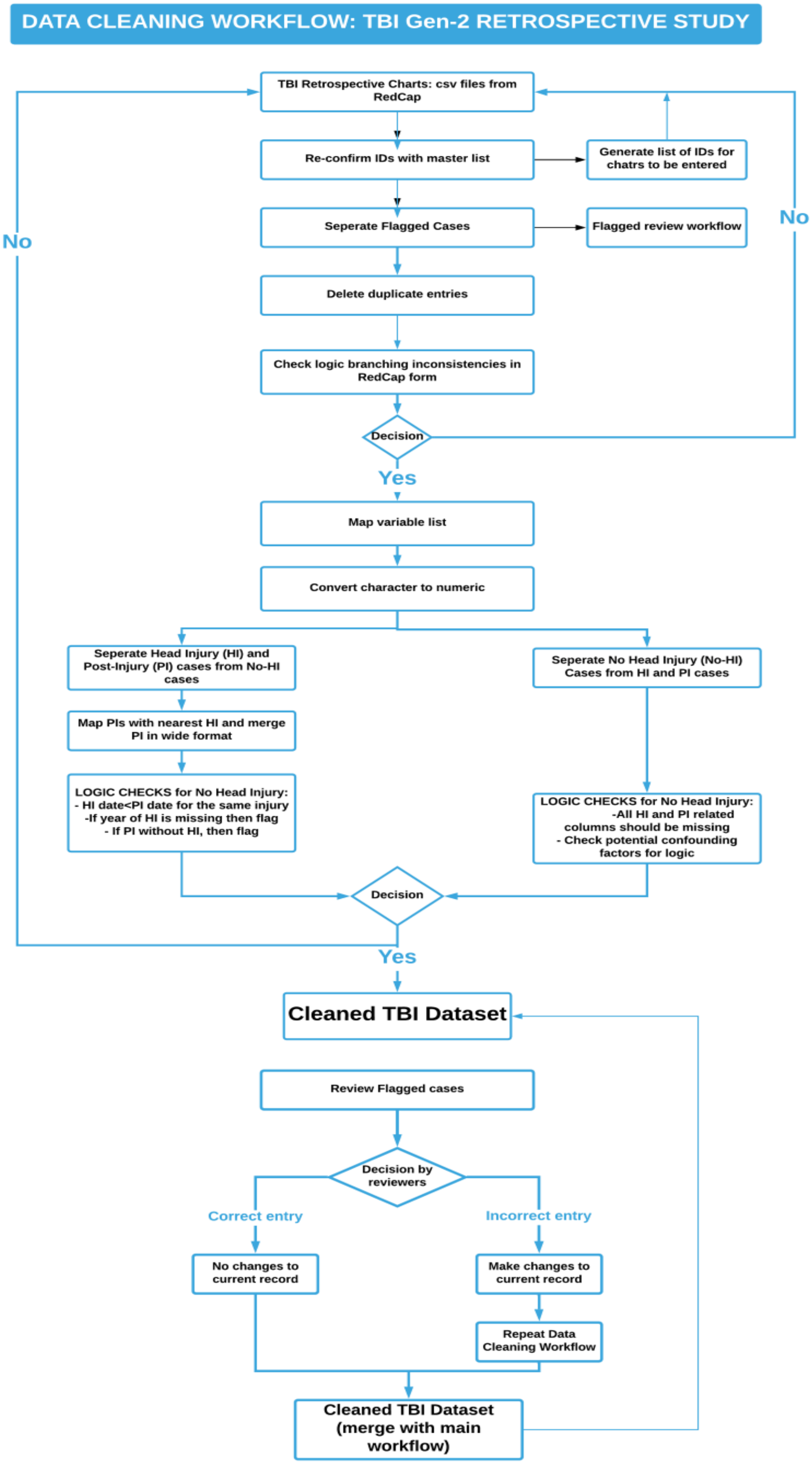

© 2026 Burton R et al. *JAMA Network Open*.

eFigure 2. Participant Selection Flow Chart for Time-to-Event Analyses Among the Nested Matched Samples in the Original and Offspring Cohorts

(A) Original Cohort

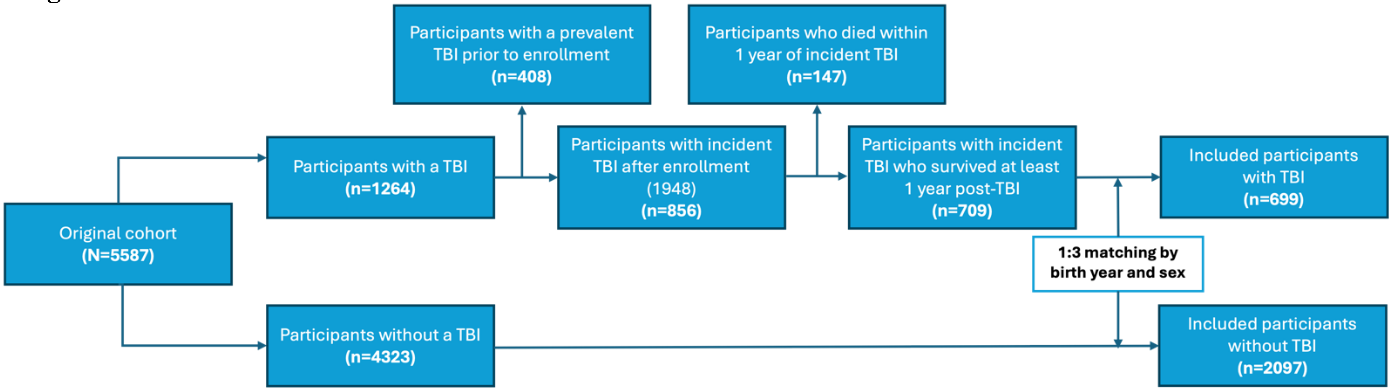

(B) Offspring Cohort

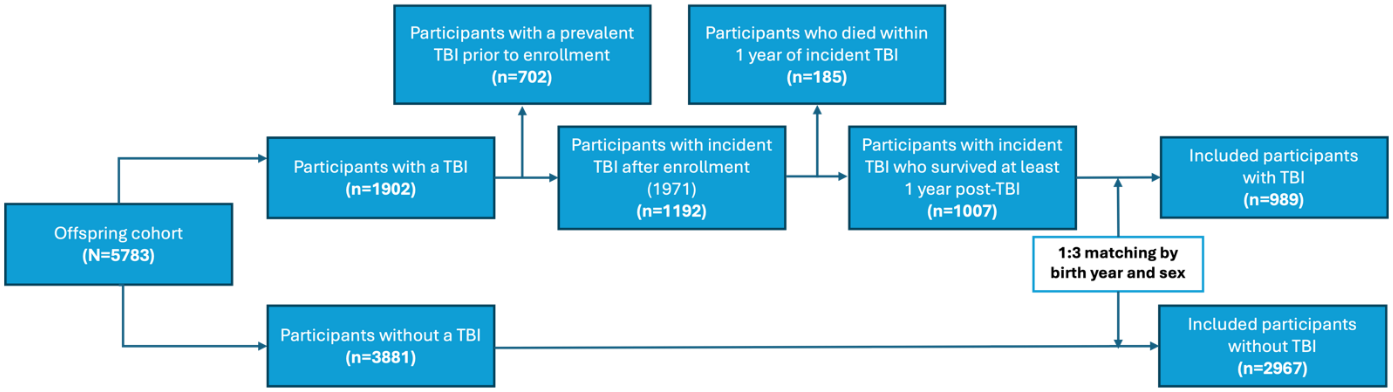

© 2026 Burton R et al. *JAMA Network Open*.

eTable 1. Modified American Congress of Rehabilitation Medicine and US Department of Veterans Affairs and Department of Defense Guidelines for Traumatic Brain Injury (TBI) Classification

Criteria	Head injury, no TBI	Mild TBI	Moderate TBI	Severe TBI
Structural Imaging	Normal	Normal	Normal or abnormal	Normal or abnormal
Loss of consciousness	None	<30 minutes	30 minutes to 24 hours	>24 hours
Altered Consciousness, Altered Mental Status, Focal Neurological Deficit	None	<24 hours	>24 hours	>24 hours
Post-traumatic amnesia	None	<1 day	1 day to 7 days	>7 days
Glasgow Coma Scale	15	13-15	9-12	3-8
Seizures after injury or both before and after injury	Absent	Present	Present	Present
Brain surgery as result of TBI	Absent	Absent	Present	Present
Died due to TBI	Absent	Absent	Absent	Present

© 2026 Burton R et al. *JAMA Network Open*.

eTable 2. Demographics by Matched Sample (n = 6752)

	TBI Status				
	TBI present (n=1688)	Mild TBI (n=1400)	Moderate-severe TBI (n=288)	No TBI (n=5064)	Total (N=6752)
Age at TBI/Initiation age	69 ± 16	69 ± 16	72 ± 15	69 ± 16	69 ± 16
Sex					
Male	677 (40.1)	556 (39.7)	121 (42.0)	2031 (40.1)	2708 (40.1)
Female	1011 (59.9)	844 (60.3)	167 (58.0)	3033 (59.9)	4044 (59.9)
Education[a]					
No High School Diploma	313 (18.5)	265 (18.9)	48 (16.7)	529 (10.5)	842 (12.5)
High School Diploma	485 (28.7)	405 (28.9)	80 (27.8)	1060 (20.9)	1545 (22.9)
Some College	361 (21.4)	298 (21.3)	63 (21.9)	1394 (27.5)	1755 (26.0)
College Degree	427 (25.3)	351 (25.1)	76 (26.4)	1712 (33.8)	2139 (31.7)
Died	1100 (65.2)	911 (65.1)	189 (65.6)	3059 (60.4)	4159 (61.6)
Cause of Death					
Dementia-related	309 (28.1)	254 (18.1)	55 (19.1)	620 (20.3)	929 (22.4)
Cardiovascular Diseases	211 (19.2)	179 (12.8)	32 (11.1)	550 (18.0)	761 (18.3)
Cerebrovascular Accident	59 (5.4)	45 (3.2)	14 (4.9)	234 (7.7)	293 (7.1)
Cancer	182 (16.6)	158 (11.3)	25 (8.7)	597 (19.5)	780 (18.8)
Other causes	204 (18.6)	161 (11.5)	43 (14.9)	611 (20.0)	815 (19.6)
Unknown	135 (12.3)	108 (7.7)	20 (6.9)	447 (14.6)	582 (14.0)
Diabetes status	193 (11.4)	158 (11.3)	35 (12.2)	408 (8.1)	601 (8.9)
Hypertension status	990 (58.7)	810 (57.9)	180 (62.5)	2697 (53.3)	3687 (54.6)
Smoking status[b]	309 (18.3)	267 (19.1)	42 (14.6)	969 (19.1)	1278 (18.9)
Stroke status	261 (15.5)	205 (14.6)	56 (19.4)	848 (16.8)	1109 (16.4)
Cardiovascular disease status	491 (29.1)	409 (29.2)	82 (28.5)	1317 (26.0)	1808 (26.8)
Age strata					
60 years and greater	1218 (72.2)	990 (70.7)	228 (79.7)	3654 (72.2)	4872 (72.2)
Less than 60 years	470 (29.4)	410 (29.3)	60 (20.8)	1410 (27.9)	1880 (27.8)

TBI: Traumatic Brain Injury
Missing data
a. Education: 471 (6.98%) missing
b. Smoking status: 79 (1.17%) missing

© 2026 Burton R et al. *JAMA Network Open*.

eTable 3. Adjusted Hazard Ratios (HRs) for the Association Between Traumatic Brain Injury (TBI) and All-Cause Mortality by Sex

	Male (N=2708)	Female (N=4044)
	HR (95% CI)	HR (95% CI)
Mild TBI	1.23 (1.06 – 1.43)	0.97 (0.86 – 1.10)
Moderate-Severe TBI	1.49 (1.08 – 2.05)	2.16 (1.64 – 2.85)
No TBI	1 (ref)	1 (ref)

Participants were matched between TBI and no TBI in a 1:3 ratio. Follow-up served as the time scale. All models were adjusted for covariates including education status, cigarette smoking, diabetes, hypertension, cardiovascular disease and stroke history.

© 2026 Burton R et al. *JAMA Network Open*.

eTable 4. Adjusted Hazard Ratios (HRs) for the Association Between Traumatic Brain Injury (TBI) and Dementia-Related Mortality by Sex

	Male (N=2708)	Female (N=4044)
	HR (95% CI)	HR (95% CI)
Mild TBI	2.56 (1.71 – 3.83)	1.38 (1.08 – 1.76)
Moderate-Severe TBI	1.88 (0.82 – 4.29)	5.07 (2.83 – 9.08)
No TBI	1 (ref)	1 (ref)

Participants were matched between TBI and no TBI in a 1:3 ratio. Follow-up served as the time scale. All models were adjusted for covariates including education status, cigarette smoking, diabetes, hypertension, cardiovascular disease and stroke history.

© 2026 Burton R et al. *JAMA Network Open*.

eTable 5. Adjusted Hazard Ratios (HRs) for the Association Between Traumatic Brain Injury (TBI) and All-Cause Mortality by Age at TBI (<60 and ≥60 Years)

	60 years and above (N=4872)	Below 60 years (N=1880)
	HR (95% CI)	HR (95% CI)
Mild TBI	1.07 (0.95 – 1.19)	1.01 (0.85 – 1.21)
Moderate-Severe TBI	1.72 (1.37 – 2.16)	2.35 (1.42 – 3.89)
No TBI	1 (ref)	1 (ref)

Participants were matched between TBI and no TBI in a 1:3 ratio. Follow-up served as the time scale. All models were adjusted for covariates including education status, cigarette smoking, diabetes, hypertension, cardiovascular disease and stroke history.

© 2026 Burton R et al. *JAMA Network Open*.

eTable 6. Adjusted Hazard Ratios (HRs) for the Association Between Traumatic Brain Injury (TBI) and Dementia-Related Mortality by Age at TBI (<60 and ≥60 Years)

	60 years and above (N=4872)	Below 60 years (N=1880)
	HR (95% CI)	HR (95% CI)
Mild TBI	1.80 (1.44 – 2.24)	0.82 (0.48 – 1.42)
Moderate-Severe TBI	3.49 (2.17 – 5.63)	6.16 (1.08 – 35.34)
No TBI	1 (ref)	1 (ref)

Participants were matched between TBI and no TBI in a 1:3 ratio. Follow-up served as the time scale. All models were adjusted for covariates including education status, cigarette smoking, diabetes, hypertension, cardiovascular disease and stroke history.

© 2026 Burton R et al. *JAMA Network Open*.

eTable 7. Adjusted Hazard Ratios (HRs) for the Association Between Traumatic Brain Injury (TBI) and All-Cause Mortality by TBI Count (n = 1617)

	HR (95% CI)
More than 1 TBI	2.00 (1.66 – 2.41)
Single TBI	1 (ref)

Participants were matched between more than 1 TBI and 1 TBI in a 1:2 ratio. Follow-up served as the time scale. All models were adjusted for covariates including education status, cigarette smoking, diabetes, hypertension, cardiovascular disease and stroke history.

© 2026 Burton R et al. *JAMA Network Open*.

eTable 8. Adjusted Hazard Ratios (HRs) for the Association Between Traumatic Brain Injury (TBI) and Dementia-Related Mortality by TBI Count (n = 1617)

	HR (95% CI)
More than 1 TBI	3.61 (2.42 – 5.39)
Single TBI	1 (ref)

Participants were matched between more than 1 TBI and 1 TBI in a 1:2 ratio. Follow-up served as the time scale. All models were adjusted for covariates including education status, cigarette smoking, diabetes, hypertension, cardiovascular disease and stroke history.

© 2026 Burton R et al. *JAMA Network Open*.

eTable 9. Adjusted Hazards Ratio (HR) for the Association Between Traumatic Brain Injury (TBI) and Cause-Specific Mortality (n = 6752)

	Mild TBI	Moderate-severe TBI
	HR (95% CI)	HR (95% CI)
Dementia-related	1.60 (1.31 – 1.97)	3.67 (2.31 – 5.80)
Cardiovascular Diseases	1.12 (0.89 - 1.39)	1.13 (0.63 – 2.03)
Cerebrovascular Accident	1.01 (0.39 – 2.63)	18.46 (2.21 – 154.34)
Cancer	0.94 (0.76 – 1.16)	1.37 (0.81 – 2.34)
Other causes	0.88 (0.78 – 0.98)	1.25 (0.97 – 1.63)
Unknown	1.10 (0.83 – 1.47)	1.29 (0.69 – 2.43)

Participants were matched between TBI and no TBI in a 1:3 ratio. Follow-up served as the time scale. All models were adjusted for covariates including education status, cigarette smoking, diabetes, hypertension, cardiovascular disease and stroke history.

© 2026 Burton R et al. *JAMA Network Open*.

eTable 10. Sensitivity Analysis Exploring Reverse Causality

(A) All-cause mortality

	HR (95% CI)	
	Sensitivity analysis, among participants with TBI prior to cognitive impairment onset and their unexposed matches	Sensitivity analysis, among participants with TBI after cognitive impairment onset and their unexposed matches
Mild TBI	1.09 (0.98 – 1.22)	0.88 (0.71 – 1.09)
Moderate-Severe TBI	1.69 (1.31 – 2.19)	1.65 (0.91– 2.99)
No TBI	1 (ref)	1 (ref)

(B) Dementia-related mortality

	HR (95% CI)	
	Sensitivity analysis, among participants with TBI prior to cognitive impairment onset and their unexposed matches	Sensitivity analysis, among participants with TBI after cognitive impairment onset and their unexposed matches
Mild TBI	1.45 (1.06 – 1.99)	0.83 (0.62 – 1.10)
Moderate-Severe TBI	3.78 (1.52 – 9.44)	1.79 (0.86 – 3.77)
No TBI	1 (ref)	1 (ref)

© 2026 Burton R et al. *JAMA Network Open*.

eTable 11. Sensitivity Analysis Using a 2-Year Threshold Between Traumatic Brain Injury (TBI) and Death

(A) All-cause mortality

	HR (95% CI)
	Sensitivity analysis, among participants who died after 2 years of TBI and their unexposed matches
Mild TBI	1.09 (0.98 – 1.22)
Moderate-Severe TBI	1.79 (1.37 – 2.34)
No TBI	1 (ref)

(B) Dementia-related mortality

	HR (95% CI)
	Sensitivity analysis, among participants who died after 2 years of TBI and their unexposed matches
Mild TBI	1.43 (1.05 – 1.94)
Moderate-Severe TBI	3.65 (1.46 – 9.15)
No TBI	1 (ref)

© 2026 Burton R et al. *JAMA Network Open*.

eTable 12. Sensitivity Analysis Stratifying Dementia-Related Mortality Into Pure Alzheimer Disease (AD)–Related Mortality and Other Dementia-Related Mortality (n = 6752)

(A) Pure AD Dementia Mortality

	HR (95% CI)
	Pure AD Dementia Mortality
Mild TBI	1.36 (0.88 – 2.11)
Moderate-Severe TBI	2.76 (0.80 – 9.54)
No TBI	1 (ref)

(B) Other dementia-related mortality

	HR (95% CI)
	"All other" Dementia Mortality
Mild TBI	2.43 (1.68 – 3.52)
Moderate-Severe TBI	3.09 (1.39 – 6.87)
No TBI	1 (ref)

© 2026 Burton R et al. *JAMA Network Open*.

eReferences.

1. Dawber, T. R., Meadors, G. F., & Moore, F. E. (1951). Epidemiological Approaches to Heart Disease: The Framingham Study. *American Journal of Public Health and the Nations Health*, *41*(3), 279–286. https://doi.org/10.2105/AJPH.41.3.279
2. Feinleib, M., Kannel, W. B., Garrison, R. J., McNamara, P. M., & Castelli, W. P. (1975). The framingham offspring study. Design and preliminary data. *Preventive Medicine*, *4*(4), 518–525. https://doi.org/10.1016/0091-7435(75)90037-7
3. Satizabal, C. L., Beiser, A. S., Chouraki, V., Chêne, G., Dufouil, C., & Seshadri, S. (2016). Incidence of Dementia over Three Decades in the Framingham Heart Study. *New England Journal of Medicine*, *374*(6), 523–532. https://doi.org/10.1056/NEJMoa1504327
4. Tsao, C. W., & Ramachandran, V. S. (2015). Cohort Profile: The Framingham Heart Study (FHS): overview of milestones in cardiovascular epidemiology. *International Journal of Epidemiology*, *44*(6), 1800–1813. https://doi.org/10.1093/ije/dyv337
5. Harris, P. A., Taylor, R., Thielke, R., Payne, J., Gonzalez, N., & Conde, J. G. (2009). Research electronic data capture (REDCap)—A metadata-driven methodology and workflow process for providing translational research informatics support. *Journal of Biomedical Informatics*, *42*(2), 377–381. https://doi.org/10.1016/j.jbi.2008.08.010

© 2026 Burton R et al. *JAMA Network Open*.
