# Supplementary material for: Traumatic Brain Injury and All-Cause and Dementia-Related Mortality in the Framingham Heart Study
Source: JAMA Netw Open. 2026 Jan 30;9(1):e2555138. doi: 10.1001/jamanetworkopen.2025.55138 (PMC12859724; doi:10.1001/jamanetworkopen.2025.55138)
Supplement: Supplement 2. — Data Sharing Statement [file jamanetwopen-e2555138-s002.pdf]

Data Sharing Statement

Burton. Traumatic Brain Injury and All-Cause and Dementia-Related Mortality in the Framingham Heart Study. *JAMA Netw Open*. Published January 30, 2026.
doi:10.1001/jamanetworkopen.2025.55138

Data
Data available: Yes
Data types: Deidentified participant data, Data dictionary
How to access data: Deidentified participant data and the associated data dictionary are available through BioLINCC at https://biolincc.nhlbi.nih.gov/home/. Additionally, requests for data access can be made directly through the Framingham Heart Study at https://www.framinghamheartstudy.org/fhs-for-researchers/ or the FHS Brain Aging Program at https://www.bumc.bu.edu/fhs-bap/contact/.
When available: With publication

Supporting Documents
Document types: None

Additional Information
Who can access the data: Data will be available upon publication to approved principal investigators.
Types of analyses: Approved analyses should result in deliverables such as manuscripts, grants, and/or training materials.
Mechanisms of data availability: Researchers seeking direct access from FHS must submit a proposal through the study's website for approval by study leaders. A data-sharing fee may apply to support administrative and technical costs.
